# Supplementary material for: Evaluation of Online Counseling through the Working Experiences of Mental Health Therapists Amidst the COVID-19 Pandemic
Source: Healthcare (Basel). 2024 Feb 19;12(4):495. doi: 10.3390/healthcare12040495 (PMC10888081; doi:10.3390/healthcare12040495)
Supplement: Supplementary file 1 [file healthcare-12-00495-s001.zip › healthcare-2827954-supplementary.pdf]

Participants' code numbers	Gender	Age	Profession	Educational Background	Years of employment	Health / Social Service
R1	F	48	Social Worker / psychotherapist	University /MSc	20	Private office
R2	F	60	Psychologist / psychotherapist	University /MA/PhD	30	Private office
R3	F	50	Consultant	University/MA	22	Social Service (public service)
R4	F	52	Psychologist / psychotherapist	University/MA	25	Private office
R5	F	32	Social Worker	University /MSc	8	Social Service (public service)
R6	F	63	Psychologist / psychotherapist	University /MSc	12	Private office
R7	F	49	Social Worker /psychotherapist	University	22	Social Service (public service) + Private office
R8	F	43	Psychologist / psychotherapist	University/MA	15	Social Service (public service) + Private office
R9	F	34	Social Worker / Sociologist	University	5	Private office
R10	F	54	Psychologist / psychotherapist	University	20	Social Service (public service) + Private office
R11	F	52	Psychologist / psychotherapist	University/MA	24	Private office
R12	F	47	Psychologist / psychotherapist	University/MA	12	Private office
R13	F	35	Psychologist / psychotherapist	University/MA	5	Social Service (public service) + Private office
R14	F	39	Social Worker / psychotherapist	University	15	Private office
R15	F	55	Psychologist / psychotherapist	University /MSc	13	Private office
R16	F	39	Social Worker / mental health counsellor	University/MA	14	Social Service (public service)
R17	F	56	Psychologist / psychotherapist	University	32	Private office

Table S1. Participants' socio-demographic characteristics

Main Themes	Subthemes	Codes	Indicative Words or Phrases
1. Evaluation of online counseling	1.1. Working experience with online counseling<br><br>1.2. Overall assessment<br><br>1.3. Advantages and disadvantages of online counseling	Imposed condition<br><br>Worked well<br><br>Benefits vs drawbacks	*had to - did not prefer*<br><br>*was effective – satisfied*<br><br>*helpful – obstacles difficult to overcome*
2. Comparing in-person and online counseling	2.1. Similarities<br><br>2.2. Differences	Same process<br><br>Distinct issues	*issues are similar*<br><br>*there is a screen*
3. Factors influencing the effectiveness of online counseling	3.1. Contextual factors<br><br>3.2. The therapeutic process	Working conditions<br><br>Process qualities	*organizational structure - communication framework*<br><br>*method matters - the therapist and the person's needs*
4. Suggestions for the future use of online counseling	4.1. A supplementary tool used with caution<br><br>4.2. Create the appropriate conditions	Add-on alternative<br><br>Development	*you have the option - additional tool*<br><br>*education - know-how and digital accessibility*

Table S2: Main themes and sub-themes
